# Coupling axonal mRNA transport and local translation to organelle maintenance and function

**DOI:** 10.1016/j.ceb.2022.01.008

**Published:** 2022-02

**Authors:** Jose Norberto S. Vargas, James N. Sleigh, Giampietro Schiavo

**Affiliations:** 1 Department of Neuromuscular Diseases, UCL Queen Square Institute of Neurology, University College London, London, UK; 2 UCL Queen Square Motor Neuron Disease Centre, UCL Queen Square Institute of Neurology, University College London, London, UK; 3 UK Dementia Research Institute, University College London, London, UK

## Abstract

Neuronal homeostasis requires the transport of various organelles to distal compartments and defects in this process lead to neurological disorders. Although several mechanisms for the delivery of organelles to axons and dendrites have been elucidated, exactly how this process is orchestrated is not well-understood. In this review, we discuss the recent literature supporting a novel paradigm – the co-shuttling of mRNAs with different membrane-bound organelles. This model postulates that the tethering of ribonucleoprotein complexes to endolysosomes and mitochondria allows for the spatiotemporal coupling of organelle transport and the delivery of transcripts to axons. Subcellular translation of these “hitchhiking” transcripts may thus provide a proximal source of proteins required for the maintenance and function of organelles in axons.

## Introduction

The morphological complexity of neurons requires precise spatiotemporal compartmentalization of gene expression and protein localization. In humans, motor neuron axons can extend beyond one meter, whereas basal forebrain cholinergic neurons, due to their complex axonal branching, possess compounded axon lengths of around a hundred meters [1]. Furthermore, organelles are transported to distal neuronal compartments away from the soma, and thus away from the main supply of mRNAs and proteins required to replenish protein complexes. Indeed, organelles, such as mitochondria and endolysosomes, require myriad protein complexes to maintain their optimal function. Thus, a critical aspect of organelle homeostasis within axons is the availability of a steady pool of mRNAs that can be locally translated to replenish each organelle's steady-state protein composition or adapt to local environmental conditions. An emerging paradigm is the tethering of mRNA-containing ribonucleoprotein (mRNP) granules directly on organelles allowing for on-demand local translation. Furthermore, translation of these organelle-tethered mRNAs in response to diverse electrophysiological and/or molecular needs within axons and dendrites may contribute to the fine-tuning of organelle function box 1.

Box 1 **Outstanding questions**

**1.** **Specificity of mRNP tethering on organelles.**
  a. Are there specific stimuli that affect the loading rate, recruitment kinetics and composition of mRNPs on mitochondria and endosomes?
  b. Is there a RBP code for the specific recruitment of certain RNAs and RBPs on a given type of organelle relative to others?
  c. Are there other, yet to be discovered, adaptor proteins for specific RBPs/mRNAs and organelle membranes that further enable the sorting and co-shuttling of mRNAs with organelles?
**2.** **Effects of organelle transport dynamics on subcellular translation and axonal health.**
  a. Do changes in transport dynamics of organelles that tether multi-functional mRNAs, have broader effects on neuronal health due to the reduction of these transcripts in distal compartments?
  b. To what extent does impaired transport of a specific organelle affect the function of another, if the mRNAs shuttled on one organelle are required for the maintenance of the other (e.g., nuclear-encoded mitochondrial genes shuttling on endolysosomes).
**3.** **Contributions of specific RNA transport mechanisms to axonal transcriptome.**
  a. Are there distinct RNA ensembles that are transported through organelle-tethered vs motor-bound axonal transport mechanisms?
  b. Is organelle-tethering the main mechanism to localize RNA in axons *en masse*?
  c. Can organelle-tethered mRNPs transfer onto motor proteins for further redistribution in axons? Conversely, can motor-bound RNPs transfer and dock onto organelles in axons?

Alt-text: Box 1

## mRNA transport in axons – multiple levels of regulation

It is estimated that ≈2500 mRNAs localize to distal compartments of neurons [2]. RNAs destined for such long-range delivery rely on *cis-* and *trans*-acting factors, which dictate their subcellular destination [3,4]. For instance, some mRNAs have 3′ untranslated region (UTR) motifs, termed ‘zipcodes’, that direct these mRNAs to axons [4, 5, 6]. In addition, UTR motifs may affect the half-life of mRNAs [7], whereas the secondary structures of RNA may contribute to their axonal localization [6]. mRNAs associate with putative RNA-binding proteins (RBPs) that control their subcellular transport, as well as stability [4,8]. For instance, TDP-43 and FUS, which are both mutated in amyotrophic lateral sclerosis (ALS), control the axonal localization of mRNAs [9]. These RBP-mRNA complexes can phase-separate into membraneless foci [10] and are capable of associating with motor proteins for transport in axons and dendrites [11,12]. Two distinct classes of microtubule-dependent motor proteins control the plus-end directed (anterograde) and minus-end directed (retrograde) axonal transport of RNA granules - the kinesins and cytoplasmic dynein, respectively [5,13,14]. Recently, reconstituted dynein motility assays revealed that the full activation of dynein, when associated with an RBP, requires the presence of RNA [12], suggesting that some distally enriched mRNAs could direct their transport back to the soma by activating dynein. Similarly, an anterograde-directed mRNP complex composed of APC bound to *β-actin* and *β-tubulin* mRNAs, along with kinesin adaptor KAP3 and KIF3 was recently reconstituted [15]. Thus, the trafficking of mRNAs to axons is modulated by motor-dependent and combinatorial processes.

## Membrane-tethered mRNP granules

**Figure 1:**
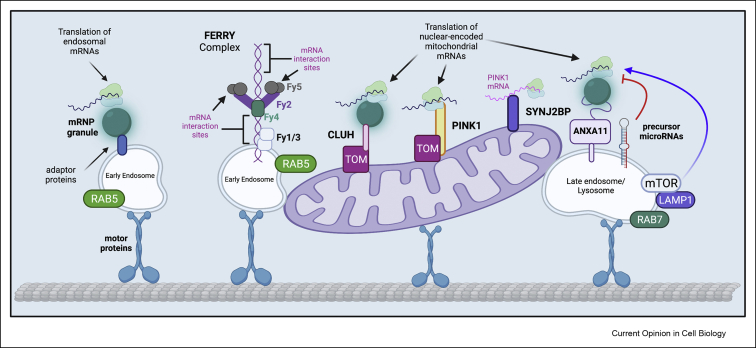
**Tethering of mRNA and ribonucleoprotein granules to organelles.** Motor proteins actively transport Rab5-positive early endosomes and Rab7- and LAMP1-positive late endosomes in axons. mRNP granules tethered to these organelles by various adaptor proteins transport mRNAs to distal compartments of neurons, facilitating *in situ* translation. Specifically, mRNPs that hitchhike on Rab5 vesicles are enriched in endosomal mRNAs, suggesting that local translation of these transcripts aids in the maintenance of endosomes in axons and dendrites. The FERRY complex is a specific adaptor protein tethering mRNAs to Rab5 containing early endosomes. It is composed of five subunits Fy1-Fy5; Fy2 holds this complex together by binding all other subunits. The coiled-coil domains of Fy2 contain various mRNA-interacting regions, which allows the FERRY complex to associate with mRNAs. Fy2 also associates directly with Rab5 to localize the FERRY complex specifically to early endosomes. Rab7-positive late endosomes, on the other hand, traffic mRNP granules via ANXA11. ANXA11 possess a membrane-binding domain that allows it to associate with LAMP1-positive vesicles, as well as an intrinsically-disordered domain that can bind mRNPs. Late endosomes also serve as hubs for precursor microRNAs. These unprocessed microRNAs can provide a pool of mature microRNAs to control the expression of mRNAs in distal compartments, and perhaps mRNAs that are tethered on late endosomes. In this manner, the transcripts, as well as their regulatory elements are co-transported on the same organelle. Moreover, the mTORC1 complex is localized and activated on the surface of late endosomes and lysosomes. Upon activation, mTORC1 initiates the translation of various mRNAs. Both Rab5-and Rab7-positive endosomes shuttle nuclear-encoded mitochondrial mRNAs, indicating that both these organelles are involved in the maintenance of mitochondria in distal neuronal compartments. Mitochondria also tether various mRNAs and mRNPs enriched for nuclear-encoded mitochondrial mRNAs. CLUH was identified to bind the outer mitochondrial membrane protein Tom20 and nuclear-encoded mitochondrial mRNAs. PINK1 kinase was also shown to bind mRNAs, linking these transcripts to mitochondria. Recently, SYNJ2BP was demonstrated to localize *PINK1* mRNA to mitochondria. The local translation of *PINK1* mRNA provides a steady pool of this protein, which is critical for mitophagy.

Apart from the mechanisms discussed above, recent studies indicate that the spatial localization of mRNPs within axons may be directly linked to organelle transport. Similar to results in a pioneering work showing cellular shuttling of mRNPs with endosomes in the fungus *Ustilago maydis* [16,17], it was recently established that mRNPs are also able to “hitchhike” on organelles in mammalian cell lines, as well as in neurons [18∗∗, 19∗∗, 20∗∗, 21∗, 22∗∗, 23∗∗], demonstrating the spatiotemporal coupling of organelle and mRNA transport. Moreover, various regulatory non-coding RNAs, such as precursor microRNAs, are enriched on endosomes and on mitochondria [24,25], suggesting that the expression of mRNAs tethered to organelles can be fine-tuned *in situ*. Local protein synthesis of these hitchhiking mRNAs may therefore contribute to the maintenance of the organelle to which they are tethered [19∗∗, 20∗∗, 21∗, 22∗∗,^26^]. A schematic summarising the studies discussed below and detailing the coupling of axonal mRNA transport and local translation to organelle maintenance and function is illustrated in Figure 1.

## Endolysosomes as hubs for mRNPs and sites of local translation within axons

One of the best examples of this emerging concept is provided by endolysosomal compartments. Endosomes are paramount for the internalization, transport, and recycling/degradation of myriad external signalling molecules and nutrients [27]. In neurons, signalling endosomes relay pro-survival signals by transporting receptor-bound neurotrophins and associated kinases from distal compartments to the soma [28]. Thus, endosomes may be an ideal platform for distributing RNAs in axons due to their bidirectional motility. A recent study utilizing image-based transcriptomics and organelle-specific RNA sequencing demonstrated the localization of various mRNAs on early endosomes [22]. Interestingly, this study also found that some mRNAs localize to early endosomes in a translation-dependent manner, as indicated by the dissociation of a pool of mRNAs from endosomes in the presence of puromycin. Furthermore, the authors found that *EEA1* mRNA, which encodes a component of early endosomes, is tethered to Rab5-positive endosomes [22]. The coding sequence of EEA1, rather than its 3′UTR region, is required for endosomal association, suggesting that non-coding regions of mRNAs are not strictly required for organelle tethering [22].

Another important study identified a novel Rab5 effector complex, termed FERRY, which binds mRNAs directly, associates with various ribosomal proteins, and tethers mRNAs to the surface of early endosomes. Thus, this complex connects early endosomes with mRNA localization and translation [23]. The FERRY complex, which is composed of five protein subunits named Fy1 to Fy5, selectively interacts with a subset of mRNAs enriched for nuclear-encoded mitochondrial genes [23]. Importantly, FERRY is present in axons of hippocampal neurons and colocalizes with both mitochondria and nuclear-encoded mitochondrial mRNAs [23]. A recent cryo-EM study showed that FERRY has a clamp-like structure, wherein Fy2 and Fy5 dimerize to form two arm-like appendages attached to a Fy4 dimer [29]. The C- and N-terminus coiled-coil domains of Fy2 contain multiple RNA-binding sites, whereas the C-terminal coiled-coil domain directly binds Fy1/3 and Rab5. Therefore, Fy2 serves as the core for other FERRY subunits, mediates mRNA binding, and tethers this complex to early endosomes [29].

Congruent with the model that endosomes shuttle mRNA along axons, it was also demonstrated that Rab7-and LAMP1-positive endosomes in axons also transport mRNPs [18,19]. In *Xenopus* retinal ganglion axons, RNA granules containing nuclear-encoded mitochondrial mRNAs were found tethered on the surface of motile Rab7-positive endolysosomes and translated on-site [19]. Strikingly, the expression of a dominant-negative Rab7 with a mutation that causes Charcot-Marie-Tooth disease type 2B (CMT2B) results in defects in endosomal trafficking, perturbs the translation of mitochondrial proteins, and diminishes mitochondrial membrane potential [19]. Interestingly, mTORC1, a kinase complex that activates translation and various catabolic processes during energy-replete states, utilizes endolysosomes as a platform for activation through specific Rag GTPases enriched on their surface [30,31]. mTORC1-dependent translation has profound effects on axonal maintenance and repair [32,33], and acts as a modifier of ALS progression [34]. These studies raise the interesting model that endolysosomes operate as supply hubs for nuclear-encoded mitochondrial mRNAs, which are then translated *in situ* to replace subunits of the electron transport chain (ETC).

Another important recent study found that RNA granules hitchhike on LAMP1-positive endosomes in axons [18]. Through proximity-based proteomics using LAMP1-APEX (ascorbate peroxidase), annexin 11 (ANXA11) was identified as an adaptor that links RNA granules to late endosomes [18]. On the one hand, ANXA11 possesses an N-terminus low complexity domain facilitating its phase-separation and association with RNA granules. On the other, its C-terminus domain binds to membranes, allowing the protein to simultaneously associate with endosomes and RNA granules [18]. ANXA11 has previously been linked to ALS [35] and ALS-causing mutations in *ANXA11* were found to disrupt its membrane-binding capacity and thus its ability to transport RNA granules in an endosomal-dependent fashion [18]. Taken together, these studies suggest an intimate link between endosomal transport, local translation, and preservation of organelle function in axons.

It is worth noting that RNA granules docked onto endosomes also carry mRNAs directly related to endosomal function, such as regulators of endocytic recycling, endocytosis, and several Rab proteins [22]. It is therefore alluring to hypothesize that the axonal maintenance of endosomes and/or their maturation require *in situ* translation of mRNAs coding for endosomal components tethered directly on the organelle. Such a mechanism augments the canonical maturation program of endosomes involving membrane remodelling and Rab conversions [36,37] by providing a source of endosomal protein synthesis in distal compartments.

## Mitochondrial maintenance in axons require co-shuttling of mRNPs and local translation

ATP synthesis, a critical function of mitochondria, requires the assembly of large multi-subunit complexes involved in the ETC. Most of the genes coding for these proteins are nuclear-encoded. Many nuclear-encoded mitochondrial mRNAs are enriched in axons and are locally translated [38,39]. The spatiotemporal coordination of both nuclear- and mitochondrial-encoded genes is intricate, especially in neurons where individual mitochondria can be meters away from the soma. One way that neurons may overcome this issue is by directly tethering mitochondrial mRNAs and RNA granules onto the organelle itself [20,21,25,26]. In this manner, the anterograde shuttling of mitochondria into axons is concurrent with the transport of mRNAs required for their repair and/or modulation of their function. Indeed, ribosomes were found to be docked on the surface of mitochondria, indicating these organelles are sites for translation [40]. In line with this, a study using proximity-specific ribosome profiling revealed that ribosomes associated with the mitochondrial outer membrane contain nuclear-encoded mitochondrial genes [41]. mRNA targeting motifs, such as 3′UTRs and mitochondrial targeting sequences, have been shown to affect not only mitochondrial localization of mRNAs, but also their translational efficiency [42]. Recent work using MS2-tagging to image *Cox7c* along live motor neuron axons demonstrated that mRNA of this essential ETC component is co-transported with mitochondria [21].

Mitochondrial maintenance involves not just replacement of organelle protein complexes, but also requires the wholesale clearance of damaged mitochondria by autophagy, a process known as mitophagy [43,44]. Mitophagy is mediated by a mitochondrion-localized kinase, PINK1 and an E3 ubiquitin ligase, Parkin [44]. Failure to induce mitophagy is a hallmark of Parkinson's disease [45]. Work in *Drosophila* showed that PINK1 also acts as a mitochondrial tether for nuclear-encoded mRNAs [26]. Furthermore, PINK1/Parkin activation dislodges translation repressors whilst activating eIF4G, a translation initiation factor [26]. Another outer mitochondrial membrane protein in flies, MDI, recruits Larp, a protein that stimulates translation of ETC subunits on the outer mitochondrial membrane [46]. Indeed, AKAP1, the human homologue of MDI, has been shown to recruit mRNAs on the surface of mitochondria [48] and was found to confer neuroprotection [47].

CLUH, a highly conserved RBP, has been shown to stabilize and foster the translation of various nuclear-encoded mitochondrial genes to regulate mitochondrial function and biogenesis [48,49]. Although CLUH is mainly cytosolic, it has also been found to tether ribosomes on the outer mitochondrial membrane through its interaction with Tom20, likely leading to co-translational import of mitochondrial proteins [50]. Recent work demonstrated that CLUH can be incorporated into G3BP-positive granules and that this process modulated mitophagy [51]. Strikingly, loss-of-function of *clueless*, the *Drosophila* homolog of CLUH, has been shown to alter neuromuscular specificity, suggesting a role in motor neuron development [52]. However, the function of CLUH in axonal biology has not been fully elucidated. Nevertheless, due to its role in localized mitochondrial maintenance by tethering mRNAs to the organelle, it is likely that CLUH plays a critical role in the upkeep of mitochondria within axons.

PINK1 has a very short half-life, in the order of minutes, and is constitutively degraded via the N-degron pathway, unless the mitochondria are damaged, which leads to PINK1 stabilization [53]. Pertinently, mitophagy occurs in axons and requires both PINK1 and Parkin [54]. However, due to the constitutive and rapid proteasomal degradation of PINK1, it was unclear how a constant supply of newly synthesized PINK1 is maintained in axons. A recent study has shed light on this issue by demonstrating that *Pink1* mRNA is tethered to mitochondria for axonal co-transport [20]. Furthermore, SYNJ2BP, a protein which has a tail-anchor domain to facilitate its mitochondrial localization, along with SYNJ2, tethers *PINK1* mRNA on mitochondria in axons [20]. Indeed, SYNJ2BP was previously identified in an unbiased screen as a putative RBP [55]. Consistently, APEX proximity labelling in tandem with protein-RNA complexes crosslinking revealed that SYNJ2BP is indeed an outer mitochondrial membrane protein capable of tethering various nuclear-encoded mitochondrial mRNAs *in situ* [56]. Consistently, loss of SYNJ2BP function inhibits mitophagy in axons and redistributes *PINK1* mRNA away from mitochondria [20]. Taken together, these studies suggest an important role for the docking of various mRNAs onto mitochondria in providing a steady supply of locally synthesized protein to maintain mitochondrial function in axons.

## Putative sorting mechanism for organelle-tethered RNAs

There are many unanswered questions regarding the packaging of mRNAs in mRNP complexes for axonal transport. It is likely that various RBPs and their numerous RNA clients form a vast overlapping network of complex interactions. Consensus binding sites of RBPs are short and degenerate [8]. Although there are many RBP motifs that were identified within transcripts, only a small subset of these is available *in vivo* due to secondary structures of mRNAs that affect RBP accessibility [57]. Moreover, several RBPs have been observed to associate with the same mRNA to control its localization. For instance, one of the most studied mRNAs within axons, *β-actin*, is bound by ZBP1, hnRNP-R, SMN, and HuD, which co-regulate its axonal and dendritic localization [58, 59, 60, 61]. FMRP granules have also been shown to colocalize with cytosolic FUS in motor neuron axons, suggesting overlap between FUS and FMRP in the mRNAs that they regulate [62]. Indeed, a large-scale yeast-two-hybrid screen for RBP–RBP interactions revealed a vast network of direct associations [63]. Importantly, by overlapping the RBP interactome with eCLIP data, it was shown that binary RBP–RBP interactions can predict combinatorial RNA binding, as well as proximal binding of interacting RBPs with mRNAs at the transcriptome scale [63]. These data suggest that the packaging of mRNPs is heterogeneous and perhaps this complexity allows RBPs to act in a cooperative manner to regulate mRNA transport in axons. On the other hand, non-classical RBPs, such as the FERRY complex, may associate with a less heterogenous set of mRNAs, since its binding does not rely on granule formation. However, as mentioned above, CLUH has been recently shown to form granules with G3BPs [51], and thus some non-classical RBPs are also able to phase-separate and incorporate with other classical RBPs.

## Organelle-coupled RNA transport – an experimental Pandora's box?

In and of themselves, organelle transport and maintenance, mRNA localization, and subcellular translation in axons, are complex biological processes [3,5]. Recent studies highlighting the interconnectedness of these processes raise the question of whether experimentally altering one perturbs the others. As an example, aberrant transport of Rab7-positive endosomes led to defects in local translation of mitochondrial genes, and in turn resulted in mitochondrial depolarization within axons [19]. One may also reasonably expect that mitochondrial transport defects could lead to a metabolic shift that alters local translation and organelle transport in axons, since both processes require ATP. Future studies are needed to demonstrate the precise links between organelle transport and subcellular translation. The emergence of proximity-based transcriptomics and proteomics [18,22,41,56] will aid in unravelling coordinated mechanisms of organelle-based mRNA transport and local translation within axons. Indeed, various neurodegenerative diseases are associated with aberrant axonal transport of organelles, as well as mutations in RBPs, and translation defects [3,9,62,64]. Thus, uncovering the interplay between these intertwined processes may yield novel therapeutic targets for ameliorating neurodegenerative disorders.

## Conflict of interest statement

Nothing declared.
